# Effects of Vornorexant, a Dual Orexin Receptor Antagonist, on Bladder Function in Rats and Nocturnal Voiding Frequency in Patients With Insomnia

**DOI:** 10.1111/iju.70547

**Published:** 2026-06-29

**Authors:** Hirohiko Hikichi, Yukihiro Chino, Teisuke Takahashi, Kazuaki Kawaura, Daiji Kambe, Noriko Hino

**Affiliations:** ^1^ Research Center, Taisho Pharmaceutical Co., Ltd. Saitama Japan; ^2^ Medical Information, Taisho Pharmaceutical Co., Ltd. Tokyo Japan; ^3^ Development Headquarters Taisho Pharmaceutical Co., Ltd. Tokyo Japan

**Keywords:** DORA, dual orexin receptor antagonist, insomnia, nocturia, vornorexant

## Abstract

**Objectives:**

Nocturia has been reported to exacerbate sleep disturbances, such as insomnia and poor sleep quality, resulting in increased risks of falls, fractures, and mortality. These consequences significantly impact the physical health and overall prognosis of patients. Dual orexin receptor antagonists, prescribed for the treatment of insomnia, have been shown to reduce nocturnal voiding frequency. However, the mechanisms underlying this effect are not yet fully elucidated.

**Methods:**

Intercontraction intervals and micturition pressure were measured using cystometry following intraduodenal administration of vornorexant to anesthetized rats. Subsequently, urine production was assessed following oral administration of vornorexant in a water‐loaded rat model. To evaluate the effect of vornorexant on nocturnal voiding frequency in patients with comorbid insomnia and mild nocturia, a post hoc analysis was conducted using data from a prior clinical study in Japanese patients. Participants were stratified by baseline voiding frequency (0 vs. ≥ 1 voids/night), and nocturnal voiding frequency was summarized.

**Results:**

In the anesthetized rats, vornorexant significantly prolonged intercontraction intervals without affecting micturition pressure. In the water‐loaded rats, vornorexant did not significantly affect urine volume. In this exploratory post hoc analysis, bedtime vornorexant significantly but modestly reduced nocturnal voiding compared with placebo in patients with ≥ 1 voids/night at baseline.

**Conclusions:**

These findings suggest that vornorexant may improve nocturia in patients with comorbid insomnia and mild nocturia, potentially through the modulation of micturition control pathways. Future clinical studies could explore the potential of improving nocturia through modulation of the micturition control pathway, which may open new possibilities for targeted therapeutic interventions.

**Abbreviations**
CMG: cystometrogram
DORA: dual orexin receptor antagonist
FAS: full analysis set
SD: Sprague–Dawley

## 1 Introduction

Insomnia is defined as a sleep disorder characterized by difficulty initiating or maintaining sleep, accompanied by daytime functional impairment [1]. On the other hand, nocturia is a condition in which individuals awaken one or more times to urinate during the main sleep period, thereby impairing quality of life [2]. It involves multiple contributing factors, including nocturnal polyuria, bladder dysfunction, and sleep disturbances [3]. Nocturia encompasses a wide clinical spectrum, ranging from mild nocturia involving sleep fragmentation to more severe forms associated with increased morbidity. Even a single nocturnal void has been associated with a higher prevalence of poor health status [4] and an elevated risk of cardiovascular disease [5].

Recent studies have reported a positive correlation between nocturnal voiding frequency and sleep [6], as well as a significant association with subjective dissatisfaction with sleep quality [7]. These findings suggest a potential bidirectional relationship between nocturnal voiding and sleep fragmentation, forming a vicious cycle. Furthermore, nocturia has been linked to increased risks of falls and fractures among older adults, indicating its impact on physical health [8]. A 5‐year prospective cohort study reported a significant association between increased nocturnal voiding frequency and higher rates of mortality, positioning nocturia as a risk factor for poor overall prognosis [9].

Orexin, consisting of orexin‐A and orexin‐B, is a neuropeptide that plays a pivotal role in the regulation of sleep and wakefulness. Emerging evidence suggests that the orexin system may also be involved in the modulation of urinary function, since several studies have reported that the dual orexin receptor antagonists (DORAs) lemborexant [10], daridorexant [11], and vornorexant [12] reduced nocturnal voiding frequency in patients with insomnia. However, the underlying mechanisms responsible for this effect remain poorly understood and warrant further investigation. Orexin‐A reportedly shortens the intercontraction intervals in the rat bladder, while the OX_1_ receptor antagonist SB334867 increases them [13]. In addition, the OX_2_ receptor antagonist TCS‐OX2‐29 has been shown to normalize shortened intercontraction intervals in the rat bladder repeatedly injected with corticosterone, with no such effects in vehicle‐treated rats [14]. Since the compound used is a selective OX_1_ or OX_2_ receptor antagonist, it is important to evaluate the effects of DORAs alone on bladder functions.

This study aimed to investigate the mechanism by which DORAs suppress nocturia. To evaluate their effects on bladder function and urine production, we investigated the effects of vornorexant on cystometric parameters and polyuria in rats using two established experimental models: cystometric assessment of bladder function in anesthetized rats, a method commonly conducted to assess bladder function [15, 16], and a water‐loaded rat model, which is used to evaluate antidiuretic effects and renal function [17]. Furthermore, we aimed to analyze the effects of vornorexant on nocturnal voiding frequency in a subpopulation of patients with insomnia who exhibited nocturnal voiding using a post hoc analysis.

## 2 Methods

### 2.1 Animals

Female Sprague–Dawley (SD) rats (age 11–12 weeks, Charles River, Yokohama, Japan) were used in the cystometry study. Male SD rats (age 8 weeks, Charles River, Yokohama, Japan) were used in the urine production study. All the animals were maintained on a 12‐h light/dark cycle in a temperature‐ and humidity‐controlled holding room, with food and water available *ad libitum*. All animal study protocols were approved by the Animal Care and Use Committee of Taisho Pharmaceutical Co., Ltd. (Protocol Nos.: PA00130, P19A0044, and P19A0047).

### 2.2 Drugs

Vornorexant hydrate, a 1/4 hydrate form of vornorexant, was synthesized at Taisho Pharmaceutical Co., Ltd. Desmopressin, a vasopressin V_2_ receptor agonist, was purchased from Prospec‐Tany Technogene Ltd. (Ness Ziona, Israel). Vornorexant was suspended in 0.5% methylcellulose, and desmopressin was dissolved in saline in a volume of 2 mL/kg for the animal studies. The dose selections for all the drugs were based on previous reports [17, 18, 19] and our preliminary studies.

### 2.3 Cystometry in Anesthetized Rats

Female SD rats were anesthetized via intraperitoneal injection of urethane (ethyl carbamate, Sigma‐Aldrich Co. LLC., USA) at a dose of 1 g/kg (0.5 g/mL) and were placed on a heating mat to maintain their body temperature throughout the measurement and recording period of cystometric parameters. Their abdomens were opened via a midline incision. The ureter was ligated and transected at a point close to the kidney. A polyethylene catheter (PE‐50, Becton‐Dickinson and Company, USA) with a fire‐flared tip was used as the bladder catheter and inserted into the bladder from the top of its dome and secured in position. To administer the dosing solution, another PE‐50 catheter, designated as the dosing catheter, was inserted and placed in the duodenum. The bladder catheter was connected via a three‐way stopcock to a pressure transducer (DX‐300, NIHON KOHDEN CORPORATION, Japan) for monitoring the intravesical pressure and to a syringe pump (TE‐331S TE‐351, Terumo Corporation, Japan) for infusing saline into the bladder. Electrical signals from the pressure transducer were amplified using an amplifier (AP‐641G, NIHON KOHDEN CORPORATION, Japan) and recorded on a PowerLab system (ML‐870, AD Instruments, USA) as cystometrograms (CMGs). All sampled electrical signals were measured using data acquisition software (LabchartPro version 7.3.7, AD Instruments, USA) at a sampling rate of 40 points/s.

To induce micturition during CMG recordings, saline was continuously infused into the bladder at a rate of 3.6 mL/h using the syringe pump. After observing at least three consecutive stable intercontraction intervals, the dosing solution was administered intraduodenally. CMG recordings continued for 60 min post‐administration under continuous saline infusion. The changes observed in intercontraction intervals and micturition pressure during the 30‐ to 60‐min period after administration were calculated as percentage changes relative to the average values of the three intercontraction intervals just before drug administration.

### 2.4 Urine Production in Conscious Water‐Loaded Rats

On the day before the test, male SD rats were transferred to the testing room and acclimated in metabolic cages under overnight fasting conditions. Vornorexant and desmopressin were administered to the rats orally 10 min prior to and subcutaneously just before the water load, respectively. Urine production was measured in terms of urine weight, assessed using urine samples collected over 2 h after administration of 30 mL/kg water in conscious rats during the light phase.

### 2.5 Assessment of Nocturnal Voiding Frequency Using a Clinical Study

The clinical trial data used for the post hoc analysis of nocturnal voiding frequency were derived from a placebo‐controlled phase 3 study conducted in Japanese patients with insomnia (NCT05453136) who received vornorexant as a therapeutic agent for insomnia [12]. The study was approved by the ethics committees of the participating medical institutions and conducted in accordance with the Declaration of Helsinki and Good Clinical Practice guidelines. All patients provided written informed consent prior to participation. The eligibility criteria have been described previously [12]; in brief, participants were Japanese men and women aged 18 years or older who had been diagnosed with insomnia according to the Diagnostic and Statistical Manual of Mental Disorders, Fifth Edition, criteria. Patients who experienced more than three episodes of nocturnal voiding per night were excluded due to concerns regarding the evaluation of sleep effect. During the screening period, patients received a placebo for the initial 2 weeks, followed by a 2‐week treatment period in which they were randomly assigned in a double‐blind manner to receive a placebo, vornorexant 5 mg, or vornorexant 10 mg. Patients recorded the number of nocturnal voiding episodes in a sleep diary that included a specific item for nocturnal voiding. The full analysis set (FAS) comprised 589 patients: 196 in the placebo group, 196 in the vornorexant 5 mg group, and 197 in the vornorexant 10 mg group. The FAS included all patients who received at least one dose of the study drug and had at least one post‐dose efficacy assessment.

### 2.6 Statistical Analysis

In the cystometry study, variance homogeneity was assessed using Bartlett's test; Shirley–Williams' test was used in cases of non‐homogeneity, and Williams' test in cases of homogeneity for multiple comparisons. For urine production, Bartlett's test was performed to assess variance homogeneity; Steel's test was used in cases of non‐homogeneity, and Dunnett's test in cases of homogeneity for multiple comparisons. In the post hoc analysis of the clinical study data, to exclude the influence of patients without nocturnal voids at baseline (Week 0), patients were stratified into two subgroups: 0 voids/night and ≥ 1 voids/night, with the latter corresponding to the clinical definition of nocturia [2]. The average number of nocturnal voids per week was rounded to the nearest integer. Categorical variables of patient background were compared using the χ^2^ test. Continuous variables were assessed using the Wilcoxon rank‐sum test for inter‐subgroup comparisons and the Kruskal–Wallis test for intra‐subgroup comparisons. Steel's test was applied for the evaluation of urinary frequency. All statistical analyses were performed using SAS software version 9.4 (SAS Institute Inc., Cary, NC, USA). A two‐tailed *p*‐value of < 0.05 was considered statistically significant.

## 3 Results

### 3.1 Cystometry in Anesthetized Rats

**FIGURE 1:**
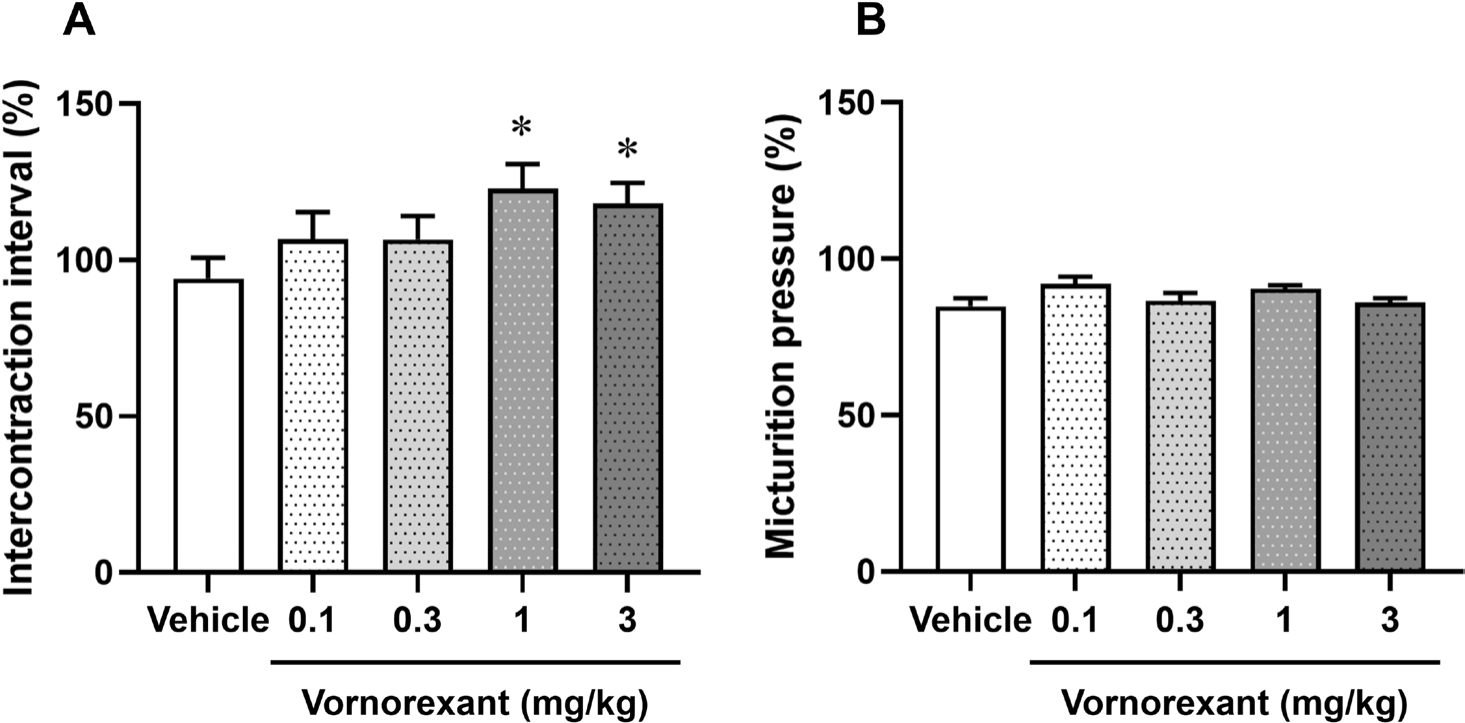
Effect of vornorexant on intercontraction interval (A) and micturition pressure (B) in cystometric analysis of the anesthetized rats. Data are presented as mean ± standard error of the mean of percentage changes relative to pre‐response (*n* = 10). *p < 0.05, compared with response in the vehicle‐treated group (Williams' test).

The intraduodenal administration of vornorexant at doses of 1 and 3 mg/kg, but not 0.1 and 0.3 mg/kg, significantly increased the percentage changes in intercontraction intervals (*p* < 0.05) (Figure 1A). In contrast, vornorexant did not affect the percentage changes in micturition pressure in the anesthetized rats (Figure 1B).

### 3.2 Urine Production in Conscious Water‐Loaded Rats

**FIGURE 2:**
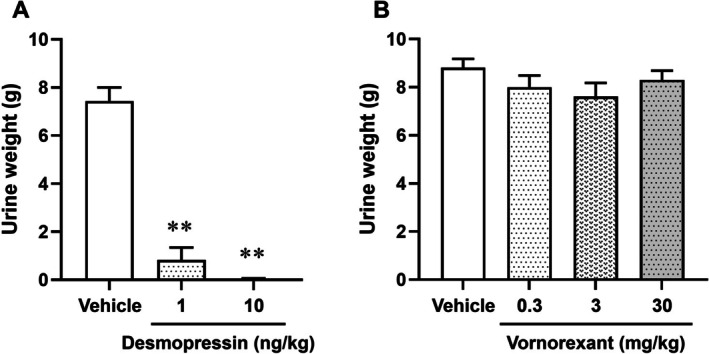
Effect of desmopressin (A) and vornorexant (B) on urine production in the water‐loaded rats. Data are presented as mean ± standard error of the mean (*n* = 8). **p < 0.01, compared with response in the vehicle‐treated group (Steel's test).

In the conscious water‐loaded rats, subcutaneous injection of desmopressin at doses of 1 and 10 ng/kg significantly decreased urine weight for 2 h after administration compared with the vehicle‐treated rats (all *p* < 0.01) (Figure 2A). On the other hand, oral administration of vornorexant did not affect urine weight for 2 h after administration (Figure 2B).

### 3.3 Demographics and Patient Background

A total of 92 (47%), 87 (44%), and 75 (38%) patients in the placebo, vornorexant 5 mg, and vornorexant 10 mg groups, respectively, had ≥ 1 nocturnal voids/night, with the mean (standard deviation) nocturnal voiding frequencies being comparable across groups at 1.2 (0.5), 1.2 (0.5), and 1.2 (0.4), respectively (Table 1). Age significantly differed between the subgroups, with patients in the ≥ 1 voids/night subgroup being older than those in the 0 voids/night subgroup (*p* < 0.001, *p* = 0.004, and *p* = 0.024 for the placebo, vornorexant 5 mg, and vornorexant 10 mg groups, respectively). For both vornorexant 5 mg (*p* = 0.017) and vornorexant 10 mg (*p* = 0.009) groups, history of hypnotic use was significantly more prevalent in the ≥ 1 voids/night subgroup than in the corresponding 0 voids/night subgroup; however, no significant differences were observed in sex or body mass index. Across the subgroups defined by 0 or ≥ 1 voids/night, no statistically significant intra‐subgroup differences were observed for any parameter (Table 1).

**TABLE 1:** Demographics and baseline characteristics.

Characteristic	Dosing group		0 voids/night	≥ 1 voids/night	Inter‐subgroup *p*‐value
Total, *n* (%)	Placebo		104 (53%)	92 (47%)	—
	VOR 5 mg		109 (56%)	87 (44%)	—
	VOR 10 mg		122 (62%)	75 (38%)	—
	Intra‐subgroup *p*‐value		0.188		
Male/female, *n* (%)	Placebo		51/53 (49%/51%)	43/49 (47%/53%)	0.748
	VOR 5 mg		53/56 (49%/51%)	41/46 (47%/53%)	0.835
	VOR 10 mg		46/76 (38%/62%)	35/40 (47%/53%)	0.215
	Intra‐subgroup *p*‐value		0.143	0.998	
Age (years), mean (SD)	Placebo		48.8 (12.1)	58.6 (11.4)	< 0.001***
	VOR 5 mg		51.4 (12.6)	57.2 (13.0)	0.004**
	VOR 10 mg		51.5 (12.1)	55.3 (13.5)	0.024*
	Intra‐subgroup *p*‐value		0.113	0.310	
History of hypnotic use, *n* (%)	Placebo		8 (8%)	13 (14%)	0.146
	VOR 5 mg		7 (6%)	15 (17%)	0.017*
	VOR 10 mg		7 (6%)	13 (17%)	0.009**
	Intra‐subgroup *p*‐value		0.837	0.806	
Body mass index (kg/m^2^), mean (SD)	Placebo		22.2 (3.6)	22.5 (3.5)	0.244
	VOR 5 mg		23.2 (3.9)	22.3 (3.2)	0.256
	VOR 10 mg		22.6 (3.9)	22.8 (3.6)	0.411
	Intra‐subgroup *p*‐value		0.120	0.818	
Nocturnal voiding frequency, mean (SD)	Placebo		0.0 (0.0)	1.2 (0.5)	< 0.001***
	VOR 5 mg		0.0 (0.0)	1.2 (0.5)	< 0.001***
	VOR 10 mg		0.0 (0.0)	1.2 (0.4)	< 0.001***
	Intra‐subgroup *p*‐value		1.000	0.684	
Nocturnal voiding frequency, *n* (%)	Placebo	0 voids/night	104 (100%)	—	—
		1 voids/night	—	76 (83%)	—
		2–4 voids/night	—	16 (17%)	—
	VOR 5 mg	0 voids/night	109 (100%)	—	—
		1 voids/night	—	71 (82%)	—
		2–3 voids/night	—	16 (18%)	—
	VOR 10 mg	0 voids/night	122 (100%)	—	—
		1 voids/night	—	65 (87%)	—
		2–3 voids/night	—	10 (13%)	—
	Intra‐subgroup *p*‐value		—	0.663	

*Note:* Categorical variables were compared using χ^2^ tests. Continuous variables were analyzed using Wilcoxon rank‐sum tests for inter‐subgroup comparisons. Kruskal–Wallis tests were applied for intra‐subgroup comparisons of continuous variables, and no significant differences were observed. *p < 0.05, **p < 0.01, ***p < 0.001.

Abbreviations: SD, standard deviation; VOR, vornorexant.

### 3.4 Nocturnal Voiding Frequency in Humans

**FIGURE 3:**
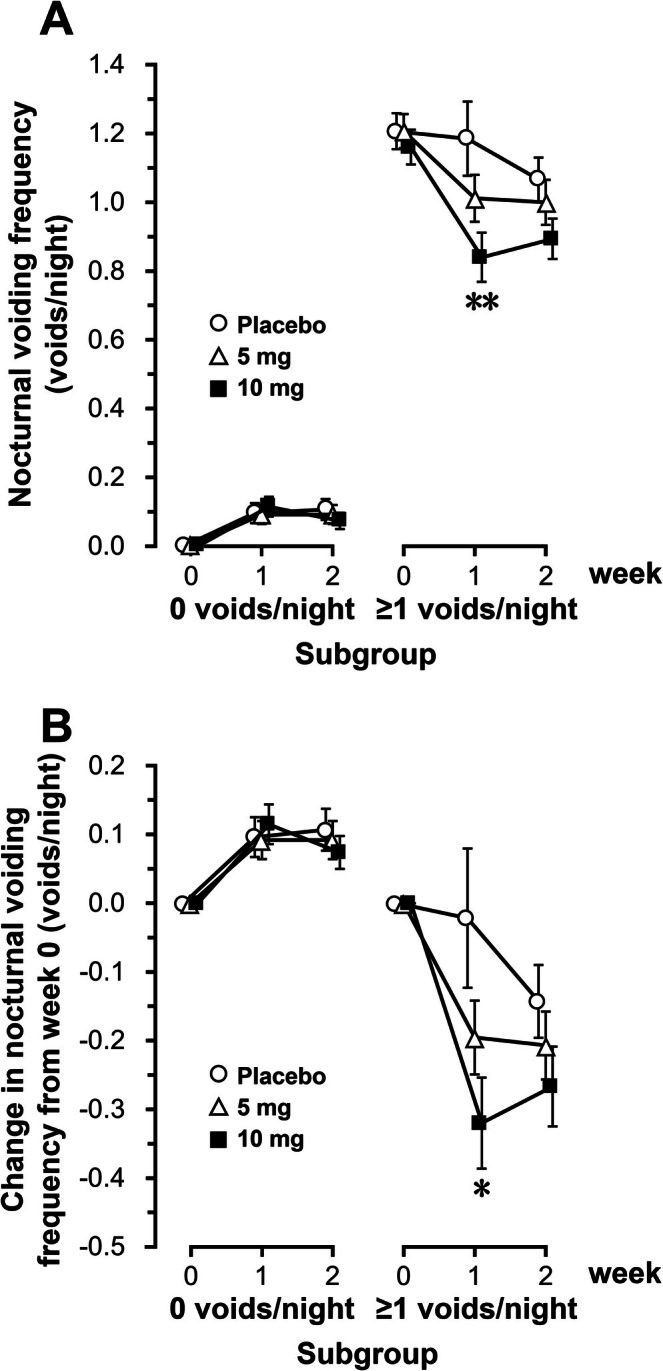
Effect of vornorexant on nocturnal voiding frequency in a subpopulation of patients with insomnia. In the post hoc analysis, participants were divided into two subgroups based on baseline nocturnal voiding frequency: 0 voids/night and ≥ 1 voids/night. The figure shows nocturnal voiding frequency (A) and changes from baseline (B). Data are presented as mean ± standard error of the mean. *p < 0.05, **p < 0.01, vs. placebo (Steel's test).

In the 0 voids/night subgroup, the mean nocturnal voiding frequency remained below 0.12 across all groups at Weeks 1 and 2, with no statistically significant differences observed (*p* > 0.59) (Figure 3A). In the ≥ 1 voids/night subgroup, nocturnal voiding frequency decreased in a dose‐dependent manner at Week 1. The mean ± standard error of the mean values were 1.18 ± 0.11 for placebo, 1.01 ± 0.07 for vornorexant 5 mg, and 0.84 ± 0.07 for vornorexant 10 mg. The vornorexant 10 mg group exhibited a significantly lower frequency than the placebo group (*p* = 0.009). This trend persisted at Week 2 (Figure 3A). For changes from baseline, in the 0 voids/night subgroup, all groups showed changes in the mean nocturnal voiding frequency below 0.12, with no statistically significant differences (*p* > 0.59) (Figure 3B). In the ≥ 1 voids/night subgroup, changes from baseline demonstrated a dose‐dependent reduction at Week 1, with values of −0.02 ± 0.10 for placebo, −0.20 ± 0.05 for vornorexant 5 mg, and −0.32 ± 0.07 for vornorexant 10 mg. A significant reduction was observed in the vornorexant 10 mg group compared with the placebo group (*p* = 0.012). This trend continued at Week 2, although no significant differences were detected between the placebo and treatment groups (Figure 3B).

## 4 Discussion

To our knowledge, this is the first study to demonstrate that the DORA vornorexant could suppress the micturition reflex without affecting micturition pressure in anesthetized rats or urine production in conscious water‐loaded rats. Moreover, in an exploratory post hoc analysis, vornorexant demonstrated a potential reduction in nocturnal voiding frequency in a subpopulation of patients with insomnia.

In the Japanese phase 3 clinical trial of vornorexant, a 10 mg dose of vornorexant significantly reduced nocturnal voiding frequency in patients with insomnia compared with placebo; however, the between‐group difference was only 0.1 episodes [12]. This limited effect may be attributed to the exclusion of patients with a nocturnal voiding frequency of > 3 voids/night, resulting in a lower baseline frequency. In the present exploratory subgroup analysis, vornorexant significantly reduced the average nocturnal voiding frequency in the ≥ 1 voids/night subgroup, demonstrating a more pronounced effect than in the overall population. In patients with insomnia, after 4 weeks, lemborexant reduced nocturnal voiding frequency from 3.4 to 2.3 episodes [10], and daridorexant reduced episodes by 1.5 and 1.6 at Weeks 1 and 4, respectively (baseline: 3.63), exceeding reductions with placebo (1.0 and 1.3, respectively) [11]. The most common adverse event associated with the OX_2_ receptor agonists TAK‐994 and danavorexton in patients with type 1 narcolepsy was urinary urgency or frequency [20, 21]. These findings suggest that the orexin system may be involved in the regulation of not only sleep and wakefulness but also urinary function and that DORAs are efficacious in both insomnia and nocturia. Regarding the temporal pattern of efficacy, in the present study, vornorexant showed a significant reduction vs. placebo at Week 1; however, this difference was no longer significant at Week 2, although a decreasing trend was maintained. A similar pattern was observed with daridorexant, which demonstrated a significant reduction at Week 1 but not at Week 4 [11]. This temporal attenuation of between‐group differences may be partly attributable to a substantial placebo effect on nocturnal voiding frequency that increases over time. However, it remains uncertain whether the improvement in nocturia observed with DORAs in clinical settings is a direct pharmacological effect or a secondary outcome resulting from the alleviation of insomnia.

To investigate the mechanism by which vornorexant reduces nocturnal voiding frequency, we examined the effects of vornorexant using animal models. In the cystometry study, vornorexant prolonged intercontraction intervals without affecting micturition pressure in anesthetized rats, suggesting that vornorexant increased the bladder capacity by suppressing the micturition reflex without increasing post‐void residual volume. Although vornorexant affected bladder functions at the same dose that produced its sleep‐promoting effect, the present study on bladder function was conducted under anesthesia. Therefore, the effects of vornorexant on bladder functions are considered to be independent of the sleep‐promoting effects. Not only bladder function, but also urine production is a key factor in nocturia. OX_1_ and OX_2_ receptor mRNAs were undetectable in the kidneys of mice [22], with extremely low OX_2_ receptor protein expression observed in the kidneys of rats [23]. Consistent with this, the present water‐loaded rat study showed no decrease in urine production following vornorexant administration. These findings suggest that the effects of vornorexant on nocturnal voiding frequency are unlikely to be mediated by changes in urine production.

The neural mechanisms underlying the blockade of the OX_1_ and OX_2_ receptors in nocturia have not yet been fully elucidated. OX_1_ receptor mRNA expression has been detected in the bladders of mice [22], while OX_2_ receptor protein expression was negligible in the bladder muscles in rats [22]. The intrathecal but not intravenous administration of orexin‐A induced the micturition reflex in rats [13], likely due to negligible brain penetration of orexin‐A in rats [24]. Therefore, these findings suggest that systemic exposure of orexin‐A does not exert direct effects on the bladder. In the central nervous system, both OX_1_ and OX_2_ receptor protein or mRNA expressions have been identified in key brain regions involved in micturition control, including Barrington's nucleus and the periaqueductal gray in rats [25, 26, 27, 28]. Immunohistochemical studies have also revealed the presence of both OX_1_ and OX_2_ receptor proteins in the spinal cord [26, 28]. As previously reported, intrathecal injection of orexin‐A shortened intercontraction intervals in rats, and this effect was attenuated by intrathecal injection of the OX_1_ receptor antagonist SB334867 [13]. Furthermore, the OX_2_ receptor antagonist TCS‐OX2‐29 has been shown to normalize the cystometric parameters associated with detrusor overactivity and to decrease c‐Fos expression in the neuronal voiding centers such as the pontine micturition center, ventrolateral periaqueductal gray, and medial preoptic area in rats repeatedly treated with corticosterone [14]. Therefore, these findings suggest that vornorexant increases bladder capacity, at least in part, by blocking orexin‐A‐induced modulation of bladder function mediated by central OX_1_ and OX_2_ receptors. However, this interpretation remains speculative, as the precise brain sites involved remain to be elucidated. To further elucidate the mechanism by which vornorexant influences nocturnal voiding, evaluation of receptor occupancy or neural activity in the brain regions and spinal cord is needed. Moreover, to translate these findings to the underlying mechanisms observed in patients, the potential influences of sleep, urine production, and voiding function in patients need to be considered. Therefore, additional clinical studies incorporating objective sleep measurements, nocturnal urine volume, and post‐void residual volume are warranted.

Some of the limitations of this study should also be acknowledged. First, although vornorexant demonstrated a direct effect on the bladder in animal experiments, the possibility of secondary effects mediated through its sleep‐promoting properties in humans cannot be excluded. Second, although subgroup sample sizes were relatively adequate (*n* ≥ 75), the analyses were not prespecified and therefore require cautious interpretation. Third, lower urinary tract symptom–related parameters were not collected, as nocturnal voiding was a secondary outcome. Although sleep‐related outcomes were assessed, they were not included because they were outside the scope of this post hoc analysis, which focused on nocturnal voiding frequency. Fourth, participants who reported > 3 voids/night were excluded, and the baseline nocturnal voiding frequency in the “≥ 1 voids/night” subgroup was relatively mild (approximately 1.2 voids/night). Therefore, the generalizability of the findings to more severe cases is limited. Lastly, nocturnal voiding frequency was rounded to an integer based on the weekly mean, which may have reduced sensitivity, and the findings were more pronounced in Week 1 than in Week 2, warranting evaluation of temporal consistency.

In summary, vornorexant is the first DORA demonstrated to modulate bladder function in rats by prolonging intercontraction intervals without affecting micturition pressure. Furthermore, post hoc analyses indicated that vornorexant has the potential to modestly reduce nocturnal voiding frequency in patients with insomnia and mild nocturia. These findings provide important evidence supporting the therapeutic potential of DORAs in the management of nocturia, which is closely associated with insomnia. Future clinical studies could explore the potential of improving nocturia through modulation of the micturition control pathway, which may open new possibilities for targeted therapeutic interventions.

## Author Contributions

**Noriko Hino:** conceptualization, formal analysis, funding acquisition, investigation, methodology, project administration, supervision, writing – review and editing. **Daiji Kambe:** writing – review and editing. **Kazuaki Kawaura:** formal analysis, investigation, methodology. **Hirohiko Hikichi:** conceptualization, formal analysis, investigation, methodology, writing – original draft, writing – review and editing. **Yukihiro Chino:** conceptualization, formal analysis, writing – original draft, writing – review and editing. **Teisuke Takahashi:** conceptualization, formal analysis, investigation, methodology, supervision, writing – review and editing.

## Disclosure

Animal Studies: All animal study protocols were approved by the Animal Care and Use Committee of Taisho Pharmaceutical Co., Ltd.

## Ethics Statement

The authors have nothing to report.

## Consent

The authors have nothing to report.

## Conflicts of Interest

This study and the original research were supported by Taisho Pharmaceutical Co., Ltd. All authors are employees of Taisho Pharmaceutical Co.Ltd.

## Data Availability

The data that support the findings of this study are available from the corresponding author upon reasonable request.
